# The Local Use of Carbolic Acid in Uterine Disease

**Published:** 1873-11

**Authors:** S. F. Starley

**Affiliations:** Fairfield, Texas


### THE LOCAL USE OF CARBOLIC ACID IN UTERINE DISEASE.

BY S. F. STARLEY, M. D., OF FAIRFIELD, TEXAS.

I wish to call the attention of the profession to what I consider one of the most important uses to which carbolic acid has been applied, viz: as a substitute for nitrate of silver and other caustics in the local treatment of inflammatory diseases of the uterus. For the past two years I have been in the constant habit of applying the pure crystalized acid to all manner of inflammatory abrasions and ulcerations of the os and cervix uteri, and of carrying it boldly into the uterine cavity, in cases of chronic catarrh of the lining membrane of that part. Every gynæcologist knows what severe pain and what copious hemorrhages frequently follow the application of nitrate of silver to the internal cavity of the womb, and how patients learn to dread the repetitions of such cauterization, although experiencing relief from them so soon as the unpleasant effects pass off.

From these disagreeable effects, the use of carbolic acid is absolutely free, while its curative effects are greater than that of any other caustic generally used for this purpose. We not unfrequently meet with cases where there is considerable enlargement of the uterine cavity, attended with frequent and profuse hemorrhages, the latter sometimes continuing nearly the entire inter-menstrual period. Now, if we apply nitrate of silver to the inflamed mucous membrane of the cavity in such a case, whether we apply it in solution or in the solid form, we are apt to find that our remedy has had the effect of greatly increasing the hemorrhage, and of throwing our patient into a severe and sometimes alarming paroxysm of pain. It is in just this class of cases that the local application of carbolic acid is of such signal service. We can apply it with perfect impunity, and instead of causing, it lessens the tendency to hemorrhage, and induces the contraction of the uterine cavity.

Being a local anæsthetic, it gives little or no pain at the moment of application, and in a very short time relieves the dragging pains of the back and hips, which is so constant an attendant upon this form of uterine disease. It produces no cicatricial contraction of the cervical canal, however freely applied to that cavity.

I have cured cases of granular vaginitis with it, after all other remedies known to me had been tried ineffectually. My manner of using it is simply to wrap the end of a Sims' silver uterine probe with a bit of cotton, taking care that it is wound sufficiently tight to prevent it pulling off. This I dip into the pure acid deliquesced by heat, and immediately apply it freely to the part to be cauterized. It produces a white film of coagulated albumen, precisely like that caused by applying the nitrate of silver. I usually make the applications once a week, and upon examining my patients have never found the slightest abrasion or breach of surface that could be ascribed to the action of the acid. Within the past two years I

have had the satisfaction of curing several of the most inveterate cases of uterine catarrh (corporeal endometritis—Thomas), in several of which cases other caustics, as the nitrate of silver, had been faithfully tried with but little advantage to the patients. But as soon as the carbolic acid was made to take the place of all other local caustics, the improvement was decided, and continued to the end of the treatment. In some cases I find it advantageous to make an occasional application of the argent. nit., of the acid nitrate of mercury. But as a general rule, I now make the carbolic acid the leading remedy in the local treatment of this class of cases. Other physicians have made trial of the remedy at my suggestion, and have obtained results equally satisfactory with myself.

About eighteen months ago, I was consulted by my friend, Dr. D. A. Cook, of Tehuacana, Texas, in reference to a case of chronic endometritis, in which nitrate of silver and various other local applications had signally failed to afford relief. In this case severe pain and copious hemorrhage would invariably follow the local use of the nitrate of silver.

I advised the Doctor to make trial of the carbolic acid after the manner above described. He did so, and the following extract is from a letter of his in reference to it, under date of March 21st, 1872.

"Your prescription is far superior to anything that I had used; it does all that you claim for it. Three cases of internal disease" (meaning inflammatory disease of the uterine cavity) "have been cured, or so greatly improved that I consider them convalescent, by your remedy, since I saw you. I feel very grateful to you for your suggestion."

My son, Dr. Wm. F. Starley, has also made use of the acid in the local treatment of uterine disease, with the most gratifying results.

In all cases the os and cervix should be brought into view with a good speculum, and the surfaces to which the acid is to be applied must be wiped clear of secretions before the application is made. This is most effectually done with a little cotton-wool, held in a speculum forceps, when the os and a portion of the cervical canal only is to be cauterized. But when the uterine cavity is the part to be touched, it is necessary to wrap a small bit of the cotton around the end of the Sims' probe, and with this the cavity is to be gently cleared of the secretions that would otherwise interfere with the acid coming into contact with the inflamed surfaces. While speaking of the speculum, I may say that I greatly prefer, and generally use that of Dr. J. Stockton Hough, first described in the *American Journal of the Medical Sciences*, and also in the *Journal of the Gynæcological Society*, of Boston, (Jan. No., 1872, figs. 6, 7.) This instrument is perfectly self-retaining, and can be used in almost any case. I have said nothing about the constitutional treatment, or of the other local measures to be resorted to while treating cases with the carbolic acid as a caustic. This, must of course, be left to the judgment of the practitioner in each case. My object has been to point out what I believe to be the great advantages of the acid over any or all other so-called caustics, in the local treatment of a class of diseases that everywhere adds so much to the sufferings of the fairest and most delicate portion of the human family.

